# *signatureSearch*: environment for gene expression signature searching and functional interpretation

**DOI:** 10.1093/nar/gkaa878

**Published:** 2020-10-17

**Authors:** Yuzhu Duan, Daniel S Evans, Richard A Miller, Nicholas J Schork, Steven R Cummings, Thomas Girke

**Affiliations:** Institute for Integrative Genome Biology, 1207F Genomics Building, University of California, Riverside, CA 92521, USA; California Pacific Medical Center Research Institute, 550 16th Street, 2nd floor, San Francisco, CA 94158, USA; Department of Pathology, University of Michigan, Ann Arbor, MI 48109, USA; Department of Quantitative Medicine and Systems Biology, The Translational Genomics Research Institute, 445 N. Fifth Street Phoenix, AZ 85004, USA; California Pacific Medical Center Research Institute, 550 16th Street, 2nd floor, San Francisco, CA 94158, USA; Institute for Integrative Genome Biology, 1207F Genomics Building, University of California, Riverside, CA 92521, USA

## Abstract

*signatureSearch* is an R/Bioconductor package that integrates a suite of existing and novel algorithms into an analysis environment for gene expression signature (GES) searching combined with functional enrichment analysis (FEA) and visualization methods to facilitate the interpretation of the search results. In a typical GES search (GESS), a query GES is searched against a database of GESs obtained from large numbers of measurements, such as different genetic backgrounds, disease states and drug perturbations. Database matches sharing correlated signatures with the query indicate related cellular responses frequently governed by connected mechanisms, such as drugs mimicking the expression responses of a disease. To identify which processes are predominantly modulated in the GESS results, we developed specialized FEA methods combined with drug-target network visualization tools. The provided analysis tools are useful for studying the effects of genetic, chemical and environmental perturbations on biological systems, as well as searching single cell GES databases to identify novel network connections or cell types. The *signatureSearch* software is unique in that it provides access to an integrated environment for GESS/FEA routines that includes several novel search and enrichment methods, efficient data structures, and access to pre-built GES databases, and allowing users to work with custom databases.

## INTRODUCTION

Genome-wide profiling technologies for mRNAs and proteins provide comprehensive recordings of biological processes. Their high-resolution can be used to distinguish cell and tissue types, and to classify dynamic cellular processes into distinct biological states such as developmental stages, defense responses to perturbagens, as well as to separate healthy from diseased phenotypes (1,2). To take full advantage of the fingerprint-level selectivity of the technology, so called Gene Expression Signature (GES) Search (GESS) algorithms are essential to accurately quantify the similarities among mRNA or protein profiles available in reference databases. With these methods one can identify similar GESs that are likely to be induced by the same or related biological mechanisms (3). This approach is analogous to the *similarity-function principle* used in many areas of biology, such as in genomics where genes with a high degree of sequence similarity are likely to share similar molecular functions. With the availability of databases containing GESs of thousands of treatments tested on many cell types, it is now possible to systematically search for genetic backgrounds, diseases, physiological conditions or small molecules inducing gene expression responses that are similar to a query GES. Both positively or negatively correlated search hits can provide insights into previously unknown connections among biological networks. For example, distinct diseases may lead to overlapping mRNA expression patterns resulting from the same or related immune response processes. Mutations inducing similar GESs may allow to functionally associate them with biological processes even if the affected genes do not share detectable sequence similarities. Similarly, drugs used for different therapeutic applications may have similar GESs due to related mode of actions (MOAs). Among other leads, this information can be used for identifying novel drug targets or for developing drug repurposing approaches (4). Ultimately, the technology has the potential to lead to the discovery of novel pharmaceutical treatments for diseases, such as for health conditions characterized by specific GESs that are anti-correlated with those of candidate drugs. Beyond these utilities, the GESS technology has a wide application spectrum for addressing fundamental research problems in biology and human health.

An important requirement for the GESS technology is the availability of reference databases containing GESs suitable for addressing specific research questions. GESs can be composed of gene sets (GSs), such as the identifier sets of differentially expressed genes (DEGs), or various types of quantitative gene expression profiles (GEPs) for a subset or all genes measured by a gene expression profiling technology. Some publications refer with the term GES mainly to GSs, or use as extended terminology ’qualitative and quantitative GESs’ (5). For clarity and consistency, this article defines GES as a generic term that comprises both GSs and GEPs (1). This generalization is important, because several GESS algorithms are introduced here that depend on reference databases containing GSs in some and GEPs in the majority of cases generated with various statistical methods. To also distinguish the queries (Q) from the entries in the reference databases (DB), they will be referred to as GES-Q and GES-DB entries in general descriptions, and as GS-Q or GEP-Q, and as GS-DB or GEP-DB in specific cases, respectively.

Three major approaches are commonly used to assemble community GES-DBs. First, they can be assembled from the results of published genome-wide expression experiments. Due to the heterogeneous nature of how result tables are organized in publications, the corresponding publication-based collections are often composed of GSs (e.g. DEGs in GS-DBs). Examples in this category include GeneSigDB, MSigDB, DSigDB and GSKB. (6–10). Second, both GS-DBs and GEP-DBs have been assembled by systematically re-analyzing genome-wide expression data from public repositories such as GEO (11,12). This reanalysis approach allows to include the corresponding numeric expression data, while also using consistent statistical methods for normalization, DEG detection and other analysis routines across studies. Third, large-scale experimental screening efforts have been used to assemble GEP-DBs, such as for a wide range of genetic and drug perturbation measurements across many cell types. These *de novo* screening efforts allow a high level of control over both experimental conditions as well as statistical analysis methods. Specific examples of GEP-DBs belonging into this third category are described in the next paragraph. Importantly, all three categories of GES-DBs are supported by the GESS methods introduced in this article.

One of the first screening-based GEP-DBs (13) was developed by Hughes *et al.* It contained GEPs of 300 diverse mutations and chemical treatments to functionally annotate both small molecules and genes in yeast. The study demonstrated that the cellular pathways perturbed by genetic modifications or small molecules can be determined by pattern matching. In mammalian biology, Ganter *et al.* generated a large-scale GEP-DB containing perturbations of several rat tissues with 600 drugs (14). They also demonstrated the utility of GEP-DBs for predicting pathological events in rats. However, these *in vivo* studies did not easily scale to larger quantities of small molecule assays mainly due to the high cost and time of performing compound screens on living animals. Lamb *et al.* generated the first large-scale mammalian cell line-based GEP-DB, called ’Connectivity Map’ or CMAP (1). Initially, it included GEPs for 164 drugs screened against four mammalian cell lines (15). A few years later CMAP was extended to CMAP2, which contains GEPs for 1309 drugs and eight cell lines. More recently, a much larger GEP-DB was released by the Library of Network-Based Cellular Signatures (LINCS) Consortium (16). In its initial release, the LINCS database contained perturbation-based GEPs for 19 811 drugs tested on up to 70 cancer and non-cancer cell lines along with genetic perturbation experiments for several thousand genes. The number of compound dosages and time points considered in the assays has also been increased by 10-20 fold. The CMAP/CMAP2 databases use Affymetrix Gene Chips as the platform for expression analysis. To scale from a few thousand to many hundred thousand GEPs, the LINCS Consortium uses the more economic L1000 assay. This bead-based technology is a low cost, high-throughput reduced representation expression profiling assay. It measures the expression of 978 landmark genes and 80 control genes by detecting fluorescent intensity of beads after capturing the ligation-mediated amplification products of mRNAs (15). The expression of 11 350 additional genes is imputed from the landmark genes by using as training data a collection of 12 063 Affymetrix gene chips (17). The substantial scale-up of the LINCS project provides many new opportunities to explore MOAs for a large number of known drugs and experimental drug-like small molecules. Complementary proteomics GES-DBs are also being developed by several community projects (18). Additional large-scale expression data and databases, where GESS applications can lead to interesting findings, consider cancer, tissue-specific, and single cell assays, such as TCGA, GTex and Single Cell Portal, respectively (19–21).

Because GESS results are usually composed of complex lists of perturbagens (e.g. drugs) ranked by their GES similarity to a GES-Q of interest, their functional interpretation is difficult with respect to the cellular networks and pathways affected by the top ranking results. In the case of drug-based GES-DBs, one can overcome this challenge by utilizing the knowledge of the target proteins of the top ranking drugs to perform functional enrichment analysis (FEA) based on community annotation systems, such as Gene Ontology (GO), pathways (e.g. KEGG, Reactome), drug MOAs, or Pfam domains. To perform this analysis, the ranked drug sets are converted into the corresponding target gene/protein sets they modulate, and then Target Set Enrichment Analysis (TSEA) based on a chosen functional annotation system is pursued. Alternatively, the functional annotation categories of the targets can be assigned to the drugs directly to perform Drug Set Enrichment Analysis (DSEA).

Currently, no one-stop software solution is available to perform the analyses outlined above in an integrated manner using a variety of GESS/FEA algorithms across several pre-built or custom GES-DBs. Previous work in this field includes web-based tools (1,16,22–24) and standalone software (3,25). Both types are usually restricted to the usage of specific pre-configured GES-DBs of limited size with insufficient options to choose among GESS methods. To address these limitations, we have developed the *signatureSearch* software. This R/Bioconductor package provides several important enhancements to the field including access to: (a) an integrated and flexible analysis environment for GESS applications; (b) a wide range of GESS methods; (c) novel enrichment algorithms for interpreting GESS results; (d) data containers, classes and accessor methods designed to scale to very large GES data sets; (e) batch query support for large-scale applications; (f) access to several large pre-built GES-DBs; as well as (g) support for searching custom GES-DBs.

The following provides a detailed description of *signatureSearch* that is structured as follows. First, we provide an outline of the most important data types and design concepts of the environment. This is followed by a description of the general analysis workflow principles of *signatureSearch*. Second, we introduce the underlying GESS and FEA algorithms, and explain the design and technical functionalities of the software. Fourth, we compare the performance of the core methods on real data, and illustrate the power of the software with a real-world use case example. Finally, we discuss the functionalities, results and future directions of this project.

A substantial amount of development effort has been invested by this project to provide efficient access to some of the largest GES-DBs that are currently available in the public domain (e.g. CMAP2 and LINCS). Since those databases are designed around chemical perturbation experiments, the following text will mainly use applications from the drug discovery field as examples. In this context it is important to emphasize that the design of *signatureSearch* is highly generic, meaning it can be used for GES-Qs and GES-DBs from many other research areas in biology or human health.

## MATERIALS AND METHODS

### Implementation

*signatureSearch* has been implemented as an open-source Bioconductor package using the R programming language for statistical computing and graphics. The affiliated data package *signatureSearchData*, provides direct access to large data sets, such as pre-built GES-DBs and annotation databases that are hosted on Bioconductor’s ExperimentHub. Both packages are freely available for all common operating systems. To optimize reusability and performance, their functions and data containers are designed based on existing Bioconductor S4 core classes. Some of the time consuming computations have been implemented in C++ using R’s C++ interface. Additional implementation details are provided in the *Software Design* section below. Up-to-date source locations and versions of data sets are provided in the vignettes and help files of the two packages.

### Data types of queries and databases

As outlined in the Introduction section, GESs of both queries and those stored in databases can be composed of GSs, or various types of quantitative GEPs for all genes measured by a gene expression technology or only a subset of them. Depending on the extent the expression data have been pre-processed, the following distinguishes four major levels, where the first three and fourth belong into the GEP and GS categories, respectively. These four levels are: (i) normalized intensity or count values from hybridization- and sequencing-based technologies, respectively; (ii) log fold changes (LFC) usually with base 2, *Z*-scores or *P*-values obtained from analysis routines of DEGs; (iii) rank transformed versions of the GEPs obtained from the results of level i or ii and (iv) GSs extracted from the highest and lowest ranks under level iii. Typically, the corresponding GSs are the most up- or down-regulated DEGs observed among two biological states, such as comparisons among untreated versus drug treatment or disease state. The order the DEG identifier labels are stored may reflect their ranks or have no meaning. When unclear, the text specifies which of the four pre-processing levels were used along with additional relevant details.

### Reference databases

The GESS algorithms and data structures provided by *signatureSearch* and *signatureSearchData*, respectively, are designed to work with most genome-wide expression data including hybridization- and sequencing-based methods, such as Affymetrix or L1000, and RNA-Seq. Currently, the pre-built GES-DBs in *signatureSearchData* include GEP data from the CMap and LINCS projects that are largely based on drug and genetic perturbation experiments performed on variable numbers of human cell lines (1,16,26). The CMap data were downloaded from the CMap project site (Version build02), and the LINCS data have been downloaded from GEO. Additional details on the content and design of these databases are provided in the Introduction of this article. In *signatureSearchData* these data sets have been pre-processed to be compatible with the different GESS algorithms implemented in *signatureSearch* (Table 1). Additional details about pre-processing routines are available in the Supplementary Section S1 as well as the package documentation. In addition, the package provides functions along with user instructions for generating custom databases that are compatible with the corresponding GESS methods. Moreover, instructions are provided how to work with other public domain GES-DBs including GS-DBs, such as MSigDB and GSKB (6–10).

**Table 1.** Categories of GESS algorithms by data types

Category	Method	Query	Database
Set-based	CMAP	GS	Rank^a^
	gCMAP	Rank	GS
	LINCS	GS	*Z*-scores^b^
	Fisher exact	GS	GS
Correlation	PCC/SCC^d^	LFC or SIG^c^	LFC or SIG

The table compares the different data types used as queries and databases by the GESS methods implemented in *signatureSearch*. The specific GEP types used by the methods are: ^a^rank transformed profiles, ^b^*Z*-scores, ^c^normalized intensities or read counts. ^d^Pearson or Spearman correlation coefficient.

### Compatibility among data types

The types of query and database GESs that can be combined in a search usually depends on the chosen GESS algorithm. To avoid incorrect selections for users, the corresponding GESS functions in *signatureSearch* enforce the usage of compatible query and database combinations. Which GES types are compatible with each search method is summarized in Table 1. The individual GESS methods are described in more detail in the following subsections.

### Overview of analysis workflow

**Figure 1.**
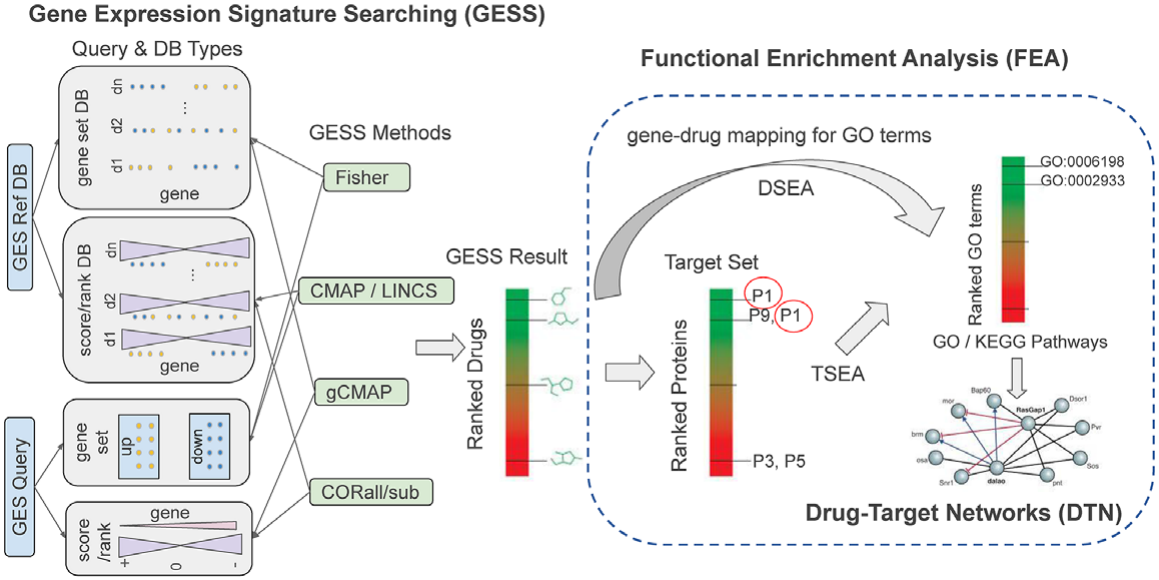
Overview of GESS and FEA workflow. GES queries are used to search a drug-based GES reference database for drugs inducing GESs similar to the query. To interpret the results mechanistically, the GESS results are subjected to functional enrichment analysis (FEA) including drug and target set enrichment analyses (DSEA, TSEA). Both identify enriched functional categories (GO terms and/or KEGG pathways) in the GESS results. Subsequently, drug-target networks (DTNs) are reconstructed for visualization and interpretation.

A typical analysis workflow in *signatureSearch* consists of three major steps (Figure 1). First, GESS methods are used to identify biological states or perturbagens such as drugs that induce GESs similar to a query GES of interest. The queries can be GSs or GEPs from genetic, drug or disease perturbations, as well as from many other experiment types. When using a GES-DB based on drug perturbations such as LINCS, then the MOAs of most drugs represented by GESs in the corresponding reference databases are known. With this information one can associate a query GES with the corresponding molecular mechanisms including available drug-target interactions. The obtained connections are useful to gain insights into pharmacological and/or disease mechanisms, and to develop novel drug repurposing approaches. Second, specialized functional enrichment analysis (FEA) methods using annotation systems, such as Gene Ontology (GO), pathways or Disease Ontology (DO), have been developed and implemented in this package to efficiently interpret GESS results. The latter are usually composed of lists of perturbagens (e.g. drugs or mutations) ranked by the GES similarity scores returned by a chosen GESS method. Interpreting these lists of perturbagens without *signatureSearch’s* functional interpretation methods is extremely difficult. Third, network reconstruction functionalities are integrated for visualizing the final results, e.g. in form of drug-target networks (DTN). Figure 1 illustrates the major steps of a typical workflow in *signatureSearch*. For each GESS and FEA step, several alternative methods have been implemented in *signatureSearch* to allow users to choose the best possible workflow configuration for their research application. Basic guidelines for choosing software tools are provided below as well as in the documentation of the package. The individual search and enrichment methods are introduced in the sections below.

For users working in drug discovery or chemical genomics, a rich suite of chemoinformatics functionalities is readily available to enhance the above workflow via the affiliated *ChemmineR* package (27,28). This way one can start with structure similarity searches to first identify related drugs represented as perturbagens in a GES database. Subsequently, the corresponding GESs are used as queries in the above GESS/FEA workflow. Moreover, one can cluster GESS results by structural or physicochemical similarities of the corresponding small molecules, e.g. to assess the quality of GESS results. The approach is based on the assumption that related compounds are more likely to induce similar GESs resulting in similar GESS rankings.

### Analysis methods

The following describes the methods used within each of the three major steps of a *signatureSearch* analysis workflow. Additional technical details about the algorithms are provided in the Supplementary Section S2 as well as in the cited source publications.

#### I. GESS methods

At the time of writing *signatureSearch* includes five GESS algorithms, with additional algorithms to be added in the future. Alternatively, users can provide their own GESS methods. Based on the data types represented in the query and database, they can be classified into set- and correlation-based methods (see Table 1 and Figure 1). The first four methods described below are set-based, whereas the last one is a correlation-based method. We refer to a search method as set-based if at least one of the two data components (query and/or database) is composed of a GS (e.g. gene labels) that may be ranked or unranked. In contrast to this, correlation-based methods require quantitative GEPs, usually of the same type for both the query and the database entries, such as normalized fluorescence intensities, read counts or Z-scores. An advantage of the set-based methods is that their queries can be the highest and lowest ranking gene sets in each direction derived from a genome-wide profiling technology that may differ from the one used to generate the reference database. However, the precision of correlation methods often outperforms set-based methods as will be shown in the Result section. This is most likely a result of the larger information content used by correlation-based methods compared to set-based methods. On the other hand, due to the nature of the expected input, correlation-based methods are usually only an option when both the query and database entries are GEPs generated by the same or at least comparable expression assay technologies. In other words, set-based methods are more technology agnostic than correlation-based methods, but may not provide the best recall performance as shown below. The following describes the most important features of each of the five GESS methods. For clarity and simplicity, the first method will be introduced in more detail, while the descriptions of the remaining ones will focus mainly on their common and unique features. Since the data types used for the queries and reference databases are different among the GESS methods, the corresponding input requirements will be specified for all of them. This is important both to understand the basic principles of the algorithms, and to choose the appropriate GESS methods for specific data sets available to users.

The Connectivity Map (CMap) GESS method (1), here termed as CMAP, uses as GS-Qs the most strongly up- and down-regulated genes from an experiment, while the reference database is composed of rank transformed GEPs (e.g. ranks of LFC or *Z*-scores) containing all genes or proteins detected by the underlying expression technology. The actual GESS algorithm of the CMAP method is based on identifying a maximum in a vectorized rank difference calculation for each of the up and down GS-Qs separately (1). After subtracting the down from the up maximum, or assigning zero to certain exceptions, the resulting raw scores are scaled to values from 1 to –1. The final ’Connectivity Scores’ expresses to what degree the up and down components of the GS-Q are enriched on the top and bottom of each database entry, respectively. The search results are a tabulated representation of the identifiers and descriptions of each GEP entry in the reference database that can be ranked by the connectivity score obtained for the corresponding GS-Q. If the utilized GEP-DB was obtained from drug perturbation experiments then the corresponding GESS scores indicate which drugs induce similar or opposing GESs as the query. Although several variants of the CMAP algorithm are available in other software packages including Bioconductor, the CMAP implementation provided by *signatureSearch* is unique by following the original description of the authors as closely as possible. This allows the reproduction of the search results obtained from the corresponding CMAP2 web service of the Broad Institute. Determining whether the results generated by both tools will consistently be the same for any GS-Q is not feasible at this point, because CMAP2 is only available as a web service that does not support large-scale queries required for systematic performance testing.

A more complex GESS algorithm was introduced by Subramanian *et al.* (16), here referred to as LINCS method. While related to the original CMAP method, there are several important differences among the two approaches. First, LINCS weights the genes in the GS-Q based on the corresponding differential expression values of the GESs in the reference database (e.g. LFC or *Z*-scores). Thus, the reference database used by LINCS needs to store the actual differential expression values rather than their ranks. Another relevant difference is that the LINCS algorithm uses a bi-directional weighted Kolmogorov-Smirnov enrichment statistic to compute a ‘Weighted Connectivity Score’ (WTCS) as similarity metric. If experimental design groups for the GEP entries in the database are available, such as shared cell types and treatment types, then the WTCS can also be normalized and standardized to obtain the ‘Normalized Connectivity Scores’ (NCS) and ‘Standardized Enrichment Scores’ (τ), respectively. To the best of our knowledge, the LINCS search and scoring functionalities in *signatureSearch* provides the first downloadable standalone software implementation of this algorithm.

The Bioconductor gCMAP (3) package provides access to a related but not identical implementation of the original CMAP algorithm described above. While the computation of the connectivity score is similar, the main difference is that gCMAP uses as a query a rank transformed GEP and each entry in the reference database is a GS composed of the labels of up- and down-regulated DEG sets. This is the opposite situation of the CMAP method, where the query is composed of the labels of up- and down-regulated DEGs and the database contains rank transformed GEPs.

Fisher’s exact test (29) can also be used as a GESS method by iteratively running the test to assess the degree of similarity shared among a GS-Q with each entry in a reference GS-DB. This method performs an over-representation analysis based on a two-by-two incidence matrix. The latter comprises set comparison counts for each GS comparison pair, including the number of genes in each GS, the numbers of their common and unique genes, the total number of genes in the reference database (universe), as well as certain derivatives of these numbers. The resulting enrichment probabilities are based on the hypergeometric distribution. To account for the multiple hypothesis testing situation of a search result, the obtained *P*-values are adjusted with the Benjamini & Hochberg method (30). In this case the search method is entirely set-based, because both the query and the database entries are composed of GSs, such as DEG sets. When the reference database is a quantitative GEP-DB then it can be converted to a GS-DB in *signatureSearch* on the fly using a user-definable cutoff (e.g. score or *P*-value).

If both the query and the database entries are available as numeric GEPs then correlation-based similarity metrics (31), such as Spearman or Pearson correlation coefficients, can be used as GESS methods. In short, correlation methods express the strength and direction of a linear relationship between two sets of paired numeric values (e.g. two GEP vectors) with a correlation coefficient. The latter is defined as the covariance of the numeric values divided by the product of their standard deviations. As non-set-based methods, they require the same type of quantitative gene expression values for both the query and the database entries, such as normalized intensities or read counts from microarrays or RNA-Seq experiments, respectively. The correlation-based searches can either be performed with the full set of genes represented in the database or a subset of them. The latter can be useful to focus the computation for the correlation values on certain genes of interest such as a DEG set or the genes in a pathway of interest. In this regard the correlation-based GESSs, performed on subsets of genes, are unique in one important aspect. That is, they allow generating meaningful GESS results for GEP-Qs, where the corresponding query genes can be derived from a variety of sources or custom collections. This means they are not necessarily expected to be the highest ranking gene or protein candidates, such as DEGs, discovered in a genome-wide profiling experiment as it is often expected for most set-based methods. The following refers to a correlation-based GESS as *SPall* or *SPsub* when considering in a search with the Spearman method the data of all assayed genes or only a subset of them (*e.g*. DEG set), respectively.

#### II. FEA methods

GESS results are lists of GEP-DB or GS-DB entries ranked by the similarity metric of a chosen GESS method. When searching drug-based GES-DBs, then the corresponding drugs are ranked accordingly. Interpreting these search results with respect to the cellular networks and pathways affected by the top ranking drugs is difficult. To overcome this challenge, the knowledge of the target proteins of the top ranking drugs can be used to perform functional enrichment analysis (FEA) based on community annotation systems, such as Gene Ontology (GO), pathways (e.g. KEGG, Reactome), drug MOAs or Pfam domains. For this, the ranked drug sets are converted into target gene/protein sets to perform Target Set Enrichment Analysis (TSEA) based on a chosen annotation system. Alternatively, the functional annotation categories of the targets can be assigned to the drugs directly to perform Drug Set Enrichment Analysis (DSEA). Although TSEA and DSEA are related, their enrichment results can be distinct. This is mainly due to duplicated targets present in the test sets of the TSEA methods, whereas the drugs in the test sets of DSEA are usually unique. Additional reasons include differences in the universe sizes used for TSEA and DSEA.

Importantly, duplications in the test sets of the TSEA are commonly caused by several distinct drugs sharing the same target proteins. Standard enrichment methods, such as those used for gene set enrichment, would eliminate these duplications since they assume uniqueness in the test sets. Removing duplications in TSEA would be inappropriate since it would erase one of the most important pieces of information of this approach. To solve this problem, we have developed and implemented in the TSEA methods of *signatureSearch* a weighting method for duplicated targets, where the weighting is proportional to the frequency of the targets in the test set.

To perform TSEA and DSEA, drug-target annotations are essential. In *signatureSearch*, they have been assembled from several sources, including DrugBank, ChEMBL, STITCH, and the Touchstone dataset from the LINCS project (16,32–34). Most drug-target annotations provide UniProt identifiers for the target proteins. If necessary, protein identifier sets can be mapped via their encoding genes to the chosen functional annotation categories, such as GO or KEGG. To minimize bias in TSEA or DSEA, often caused by promiscuous binders, it can be beneficial to remove drugs or targets that bind to large numbers of distinct proteins or drugs, respectively. To conduct TSEA and DSEA efficiently, *signatureSearch* and its helper package *signatureSearchData*, provide several convenience utilities along with drug-target lookup resources for automating the mapping from drug sets to target sets to functional categories (Table 2). To avoid additional duplications caused by many-to-one relationships among protein isoforms and their encoding genes, most FEA tests involving proteins in their test sets are performed on the gene level in *signatureSearch*. For this, the corresponding functions in *signatureSearch* will usually convert target protein sets into their encoding gene sets using identifier mapping resources from R/Bioconductor, such as the *org.Hs.eg.db* annotation package. Because of this as well as simplicity, the following text and the corresponding documentation of the software will refer to the targets of drugs almost interchangeably as proteins or genes, even though the former are usually the direct, and the latter only the indirect, targets of drugs, respectively.

**Table 2.** List of important functionalities provided by *signatureSearch* and *signatureSearchData*

Function name	Description	Input^a^	Output and comments
*(I) GES Databases*			
CMAP2	Affymetrix drug signatures	Raw, normalized and rank-based expression data	GES reference DB stored as HDF5 that
LINCS	L1000 drug & genetic signatures	Normalized and weighted averaged expression data	can be accessed via *signatureSearchData*
Custom	User provided signatures	Many types of expression data	from *ExperimentHub* or user system
*(II) GESS Methods*			
*gess_cmap*	CMAP method (1)	GS-Q: DEG; GEP-DB: *Z*-score/LFC ranks	*gessResult* object containing search result
*gess_lincs*	LINCS method (16)	GS-Q: DEG; GEP-DB: *Z*-scores	table with similarity scores for each
*gess_gcmap*	gCMAP method (3)	GEP-Q: *Z*-score/LFC ranks; GS-DB: DEG	perturbagen GES in the reference database,
*gess_fisher*	Fisher’s exact test (29)	GS-Q: DEG; GS-DB: DEG	the query signature itself, as well as details
*gess_cor*	Correlation methods (31)	GEP-Q; GEP-DB: same genes and GEP type	about the chosen search parameters
*(III) FEA Methods*			
*tsea_mGSEA*	Modified GSEA algorithm (36)	Score ranked target list	*feaResult* object containing statistical
*tsea_dup_hyperG*	Duplication adjusted hyperG test (35)	Target set with duplication	enrichment results, details about chosen
*tsea_mabs*	meanAbs method (37)	Score ranked target list	functional annotation system, labels of
*dsea_hyperG*	Hypergeometric test (35)	Drug set	drugs used for testing, as well as
*dsea_GSEA*	GSEA algorithm (36)	Score ranked drug list	their corresponding target information
*(IV) Visualization*			
*gess_res_vis*	GESS result visualization	*gessResult* object	Dot plot of drug similarity scores
*comp_fea_res*	FEA result comparison	List of *feaResult* from FEA methods	Dot plot comparing result consistency
*dtnetplot*	Drug-target networks	Drug set; pathway ID	Interactive network graph

The names of functions and libraries are italicized. ^a^Only the most common input types are listed. Acronyms are defined in the text.

The following introduces the functionalities in *signatureSearch* for performing TSEA on drug-based GESS results using as functional annotation systems GO and KEGG pathways. For this the enrichment tests can be performed with three widely used algorithms that have been modified in *signatureSearch* to take advantage of duplication information present in the test sets used for TSEA. The relevance of these target duplications is explained above. To account for multiple hypothesis testing situations, the FEA functions support seven *P*-value adjustment methods. The Benjamini & Hochberg (BH) method is usually set as the default adjustment. The latter is used for the FEA tests included in this article (30). First, we developed the *Duplication Adjusted Hypergeometric Test* (dup_hyperG). This test is based on the hypergeometric distribution, which determines whether a discovered gene set shows an enrichment in functional annotations that is more extreme than what is expected from random sampling from the same gene universe (35). To maintain the duplication information in this test, the size of the test set and number of proteins belonging to an annotation category (e.g. GO term) are both adjusted by the frequency of the target proteins in the test set. Effectively, the approach removes the duplications, but maintains their frequency information in form of weighting values. Second, we developed the *Modified Gene Set Enrichment Analysis* (mGSEA). The original GSEA method calculates the degree to which annotation categories are enriched at the extremes of ranked gene lists. For this an enrichment score is computed with a running sum Kolmogorov-Smirnov statistic and then evaluating significance by comparing the results to a null distribution derived from random queries (36). To perform GSEA with duplication support, we are introducing in *signatureSearch* a modified GSEA (mGSEA) method, where the frequency information of targets is preserved by a weighting approach. More details on the mGSEA method are provided in Supplementary Section S2. Third, we have implemented the MeanAbs (mabs) method in *signatureSearch*. MeanAbs is a simple but effective method for performing gene set-based enrichment analysis (37). It assesses the enrichment of genes in an annotation category simply by averaging their absolute values of a chosen statistics (e.g. log_2_ ratios or *Z*-scores). Subsequently, significance is evaluated by comparing the result to a null distributions derived from random permutations of queries.

Instead of translating ranked lists of drugs into target sets, as for TSEA, the functional annotation categories of the targets can be assigned to the drugs directly to perform Drug Set Enrichment Analysis (DSEA) instead. Since the drug lists from GESS results are usually unique, this strategy overcomes the duplication problem of the TSEA approach. This way the above described enrichment methods, such as GSEA or tests based on the hypergeometric distribution, can be readily accommodated in the underlying statistical methods without major modifications. As explained above, TSEA and DSEA performed with the same enrichment statistics are not expected to generate identical results. Rather, they often complement each other’s strengths and weaknesses.

#### III. DTN visualization

After identifying in drug-based GESS results enriched target classes via the above described FEA methods, it is important to visualize the results in graphical representations that are designed to simplify the functional interpretation of the analysis outcomes. To address this important need, *signatureSearch* provides functions to render the final results in form of interactive drug-target network representations.

In addition to network graphics, the *signatureSearch* package provides several other visualization and plotting functionalities. This includes visual summaries of GESS ranking scores (Table 2) which can be applied to selected perturbation types in GESS results across cell types along with cell type classifications, such as normal and tumor cells. In addition, various visualization functionalities for FEA results are available, such as dotplots and gene-concept networks. To maximize shareability and extendability across open-source environments, visualization resources from other packages are integrated such as *clusterProfiler* (38).

### Software design

Integrating analysis software for GESS and FEA applications into an R/Bioconductor package has several advantages. First, Bioconductor provides access to a large number of high-throughput genome analysis tools that are interoperable by sharing the same data structures and S4 classes optimized for statistical analysis. Second, the approach simplifies the development of automated end-to-end workflows for conducting GESSs for many application areas. Third, it consolidates an expandable number of GESS and FEA algorithms into a single environment that allows users to choose the most appropriate methods and parameter settings for a given research question. Fourth, the usage of generic data objects and classes improves maintainability and reproducibility of the provided functionalities, while the integration with the existing R/Bioconductor ecosystem, such as the widely used summarizedExperiment class infrastructure, maximizes their extensibility and reusability for other data analysis applications. Fifth, it provides access to several community perturbation reference databases along with options to build custom databases with support for most common gene expression profiling technologies (e.g. microarrays and RNA-Seq).

**Figure 2.**
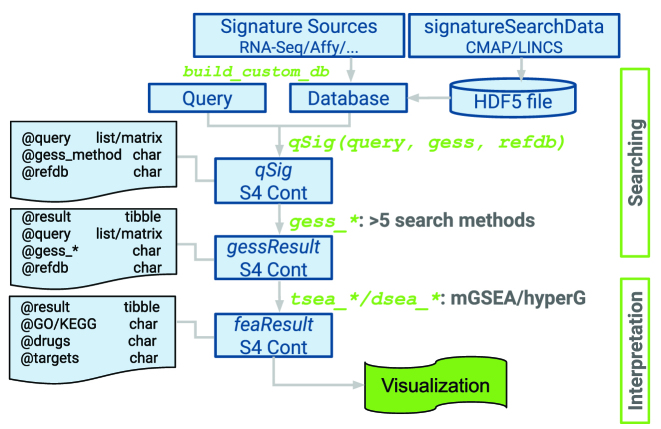
Design of *signatureSearch* package. GES reference databases are constructed from expression profile collections (RNA-Seq, Affymetrix chip or other technologies) and stored as HDF5 files. To perform GESSs, all query parameters are defined in a *qSig* search object where users can choose among over five search algorithms. The results are stored in a *gessResult* object that can be functionally annotated with different TSEA and DSEA methods. The enrichment results are organized in an *feaResult* object that can be used for drug-target network analysis and visualization.

Figure 2 illustrates the design of the package with respect to its data containers and methods used by the individual GES analysis workflow steps. Briefly, expression profiles from genome-wide gene expression profiling technologies (e.g. RNA-Seq or microarrays) are used to build a reference database stored in the Hierarchical Data Format 5 (HDF5). HDF5 is a technology that enables storage and efficient retrieval of very large data sets. For convenience the *signatureSearchData* package provides pre-built HDF5 reference databases for users. A search with a query signature against a reference database is initialized by declaring all parameter settings in a qSig search object. Currently, users can choose here among five different search algorithms implemented in *signatureSearch*, while additional algorithms will be added in the future. The five implemented algorithms are listed in Table 2 and described in the previous section of this article. To minimize memory requirements and improve time performance, large reference databases are searched by sequential or parallel processing of its GES entries in batches of user-definable size. The search results are stored in a gessResult object that contains all information required to be processed by the downstream functional enrichment analysis (FEA) methods such as drug set and target set enrichment analysis (TSEA and DSEA) methods. The resulting functional enrichment information is organized in an feaResult object that can be passed on to various drug-target network construction and visualization methods implemented in *signatureSearch*.

## Results

### Performance comparisons of GESS methods

#### Test design

Compounds with similar functional or structural properties are expected to induce GESs that are more similar among each other than those induced by compounds with dissimilar properties (16). Based on this proof-of-concept assumption, we aim to systematically compare the performance of the six GESS methods, currently implemented in *signatureSearch*, in recovering both functional and structural categories using known MOA categories and structure similarity clusters (SSC), respectively. That is, we ask the question: do drugs with similar molecular effects or structural features cluster in GESS results according to the corresponding classifications?

The MOA annotations used for these tests were downloaded from the Touchstone database (16). These include 276 MOA categories and drug-target annotations for 1555 drugs. Since not all of the MOA categories are expected to perform equally well in GESS performance tests, the MOAs were ranked by their recall rates, and 25% of the top performers (here 69 MOAs with 309 drugs) were used for testing. To avoid bias in the final MOA selection, the recall rates were calculated across all GESS methods. Additional details on this filtering procedure are provided in Supplementary Section S3. Examples of poor performing MOA categories include those enriched in drugs that bind to sets of unrelated target proteins, or drug targets positioned far downstream of transcriptional regulation processes. In both cases, the drugs of the corresponding MOA categories are not expected to induce related expression changes. Thus, including these problematic MOAs in recall performance tests would unnecessarily degrade the overall performance of the GESS methods.

The SSC categories were generated with the binning clustering method of the *ChemmineR* package (27). This clustering step used atom pairs for structure similarity comparisons and the Tanimoto coefficient as similarity metric. For assigning compounds to clusters, a Tanimoto coefficient of 0.6 was used as similarity cutoff. The latter was chosen because it often generates, in combination with the atom pair method, clusters of reasonable size with relatively low numbers of false negatives and positives (28,39). Since PC3 cells had the best screening coverage in the LINCS database, the 5253 compounds participating in the corresponding assays were used to generate the SSCs. Subsequently, the SSCs were filtered the same way as the MOA categories above, meaning only 25% of the top performers (here 139 SSCs with 542 compounds) were used for testing.

**Figure 3.**
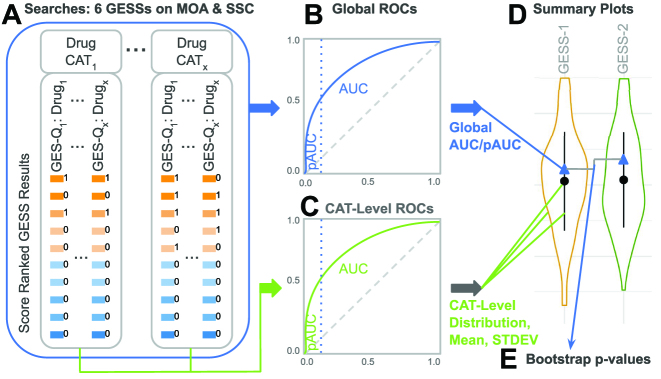
Performance testing strategy of GESS methods. (**A**) The GESs of the drugs in each MOA and SSC category were searched against the LINCS database with each of the six GESS methods. The results were sorted by the corresponding similarity scores, here indicated by boxes with color gradient. GESs from the same and different MOA/SSC categories (CAT) as the query were indicated in a binary vector with ones and zeros (next to boxes), respectively. After joining the binary vectors for each category group and re-sorting them by the corresponding scores, cumulative TPRs and FPRs were plotted in form of ROCs. This was done on the global level (**B**) and the CAT level (**C**) for the MOA and SSC classifications separately. (**D**) The distributions of AUC/pAUC values from each CAT-level are depicted by violin plots with mean values and standard deviation (STDEV) bars given in the middle. In addition, the global AUC/pAUC values are indicated by triangles. (**E**) The statistical significance of the observed differences among the global AUC/pAUC values of the six GESS methods was assessed by a bootstrap test described in the text.

The GESs induced by the drugs in each MOA and SSC category were queried with each of the six GESS algorithms against the LINCS database and their similarity scores recorded for the corresponding database entries (Figure 3 A). The query GESs of each drug used for the four set-based methods (*CMAP*, *gCMAP*, *Fisher* and *LINCS*) and the two correlation-based methods (*SPsub* or *SPall*) were the GSs corresponding to the 150 most strongly up- and 150 most down-regulated DEGs, and the GEPs subsetted to the same GSs or those for all assayed genes, respectively. The cell type, treatment time point and concentration chosen for these experiments were PC3, 24 h and 10 μM, respectively. Subsequently, the performance among GESS methods was compared in the form of receiver operating characteristic (ROC) curves by evaluating the true positive rate (TPR) against the false positive rate (FPR) across the full range of similarity scores obtained for each GESS method (40). ROCs were computed for each GESS method by calculating their cumulative TPRs and FPRs from a binary vector that was sorted by the similarity scores of the combined query results (Figure 3B and C). In each binary result component, drugs from the same and different categories as the corresponding query were indicated with ones and zeros, respectively. The same ROC calculations were performed on MOA and SSC categories separately. In both cases this was done on both the category level and the global level by generating ROCs for each category separately and all of them combined, respectively. To quantitatively compare the ROC performance results, we calculated the Area Under the Curve (AUC) as well as partial AUCs (pAUCs). While the full AUC evaluates the performance over the entire range of GESS similarity scores, the pAUCs are used for testing early enrichment at specific FPRs, where we chose FPRs of 1%, 5% and 10%. To assess whether the observed performance differences are statistically significant for all pair-wise comparisons among AUCs and pAUCs (Figure 3 D-E), the bootstrap method from Robin *et al.* (40,41) was used combined with the Benjamini & Hochberg (BH) method for multiple testing correction (30). The results of these tests are provided in Supplementary Tables S2 and S3.

#### Test results

**Figure 4.**
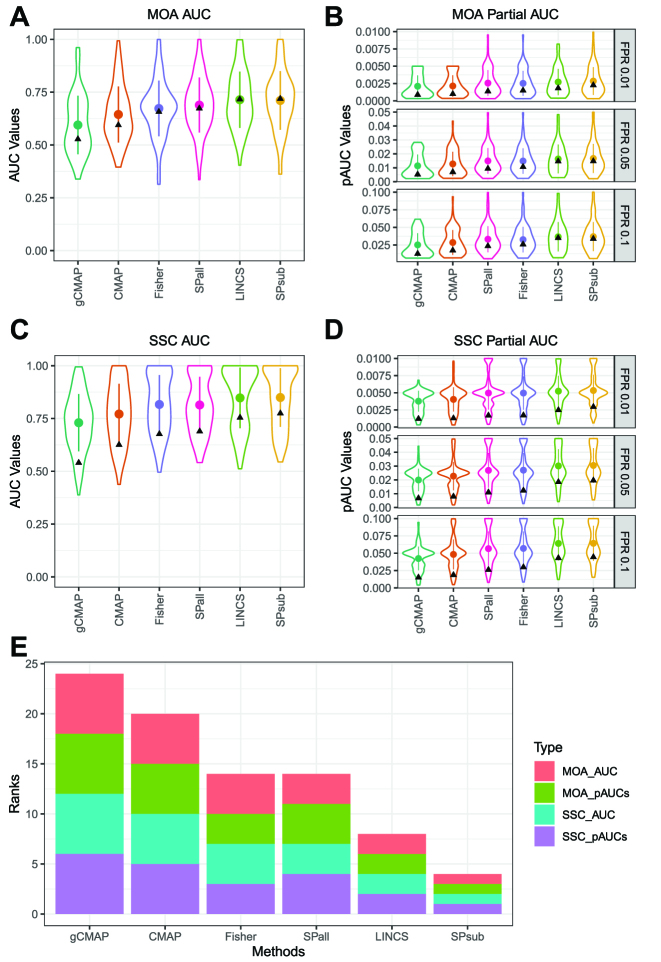
Recall performance of GESS methods on MOA and SSC categories. (**A**) The distributions of the ROC performance results of the 69 MOA categories are plotted in form of violin plots for each of the six GESS methods. The corresponding mean values, standard deviation bars and global AUCs are indicated within each violin by dots, vertical lines and triangles, respectively. The GESS methods are ordered by increasing global AUC values. (**B**) The corresponding distributions of pAUC values are given for FPRs of 1%, 5% and 10%. In this composite plot, the GESS methods are ordered by the mean of the ranks of their global pAUC values. (**C**–**D**) The GESS performance results of the 139 SSC categories are plotted the same way as the corresponding MOA results. (**E**) The performance results under (A)–(D) are summarized in form of stacked bar plots where the sum of the ranks is used to order the GESS methods from left to right by increasing performance. Each bar is composed of the ranking of the global AUCs and the mean ranking of the corresponding pAUCs for both MOA and SSC categories.

The distributions of the category level AUCs and pAUCs for MOAs and SSCs are shown in Figure 4A–B and C–D, respectively, in the form of violin plots that are sorted by the corresponding global AUC and pAUC values. Figure 4 E summarizes the performance test results for MOA and SSC categories in form of ranks of AUC and averaged pAUC outcomes. The sums of the ranks (here height of stacked bars) reflect the final performance ranking of each GESS method.

According to the performance results in Figure 4, *SPsub* consistently shows the best recall performance for MOA and SSC categories with respect to both global and early enrichment. *LINCS* performs second best for the same performance metrics. The performance rankings of the other four GESS methods are also relatively consistent across the four AUC/pAUC metrics. Their final rankings in decreasing order are: *SPall*, *Fischer*, *CMAP* and *gCMAP* (4 E). The corresponding bootstrap test results in Supplementary Tables S2 and S3 indicate that the observed differences among the AUC and pAUC values are statistically significant for nearly all pair-wise comparisons.

Among the correlation-based methods, *SPsub* performs better than *SPall* with respect to the AUC and pAUC performance metrics. One reason for this trend may be a lower noise level in the expression profiles used for computing the correlation coefficients for *SPsub* than *SPall*. The GEPs used for the *SPsub* method are usually enriched in genes (here most up- and down-regulated DEGs) that are robustly expressed, whereas the full gene repertoire used by *SPall* contains a larger proportion of genes with noisy expression signals. Among the set-based GESS methods, LINCS performs best, while the classical Fisher’s exact test outperforms *CMAP* and *gCMAP* with respect to AUC and pAUC metrics for both MOA and SSC categories. The stronger performance of the *LINCS* method compared to the other three set-based methods is most likely due to the additional weighting information utilized by this method.

Importantly, the global AUC values of the GESS methods are not expected to be very close to the best possible value of 1. However, they are in a high enough range to be substantially distinct from random assignments of drugs to MOA and SSC categories. In panel A and C of Figure 4, the global AUC values for MOA and SSC categories range from 0.53 to 0.72 and 0.54 to 0.77 with mean values of 0.65 and 0.68, respectively. It also has to be noted that the AUCs of the SSC categories are consistently higher than their MOA counterparts. This trend is expected because the SSCs were assembled with a single algorithm resulting in more homogeneous compound categories than the more complex annotation-based MOA classification system.

While the ranges of the AUC values for both classification types are reasonably high for real data, their absolute values should not be confused with a metric suitable for judging how well the majority of the GESS methods or the underlying GES assay technologies perform overall. Lower AUC values are expected for both category types mainly due to the complex nature of the real data set used for testing without degrading the reliability of the AUC-based ranking of the GESS methods. Clearly, the statistically significant ranking of the AUC values is the relevant information obtained from these tests. The following gives additional details why the maximum achievable AUC values are expected to be lower. First, the chosen MOA classifications are based on complex drug annotation data, which often do not have simple and unambiguous ground truth answers as it is possible with synthetic data. The structural similarity groupings of the SSC categories are also not expected to join compounds into groups, where every member is guaranteed to interact with the same targets or molecular processes. Second, the high noise level present in real large-scale mRNA expression data make correct assignments challenging, which in turn causes an additional reduction of the AUC values. Third, the presence of drugs binding to several targets from different MOA and SSC categories induces complex composite GESs. Finally, categories far up- or downstream of transcriptional control processes are unlikely to contain many drugs that recall each other to a high degree, no matter how well a GESS method performs overall. Despite these limitations, the MOA- and SSC-based GESS performance testing methods, chosen for this study, are appropriate choices in this use case, because they capture more biologically relevant information than alternative classification approaches based on synthetic data.

#### Time and memory performance

The GESS methods in *signatureSearch* process reference databases in batches with user-definable numbers of GES entries in each iteration of a full database scan. This allows searching of very large databases, while capping the memory consumption within the resources available on a computer system without major compromises on time performance. The time and memory performance of the six GESS methods is given in Table 3 for searching the LINCS database subsetted to ten thousand entries with a batch size limit of five thousand. The differences among the methods with respect to memory footprint and time performance for a fixed batch size is largely proportional to the size differences of the data required for each algorithm. For instance, the methods *SPsub* and *Fisher* only require for each GES entry the GSs of the most up- and down-regulated genes, whereas CMAP, LINCS and SPall require quantitative or rank-transformed GEPs for all assayed genes. Similarly, the processing times are shorter for the methods with more compact database entries, due to shorter load times when reading batches of GES entries into memory. The above time performance results are given for a single CPU core. If additional performance is needed (e.g. with very large databases), then it is easy to accelerate the search times by using the parallelization routines available in R/Bioconductor, such as *BiocParallel* or *batchtools* (42).

**Table 3.** Time and memory performance

GESS method	Time	Memory
CMAP	1.2 min	3.5GB
LINCS	1.7 min	2.3GB
gCMAP	1 min	290MB
Fisher	9 s	238MB
SPall	1 min	838MB
SPsub	13 s	238MB

#### Comparisons with competing software

This project implements commonly used GESS methods in a single environment including those that were previously only available as web services. Their performance has been compared above (Figures 3 and 4). Direct comparisons with web services are not an option for these tests, because they require large scale queries in the range of thousands of database searches with control over the GES database composition. Those requirements are usually not supportable by web services.

### Use case

The following demonstrates how the functionalities of *signatureSearch* can be applied to discovery-oriented research related to basic questions in biology, drug discovery and biomedical sciences. We selected as a query the GEP of SKB cells (skeletal muscle forming myoblasts) treated with vorinostat to search the LINCS expression database with the *SPsub* method. The latter GESS method was selected because it produced the strongest results in the above performance tests (Figures 3 and 4). Both the query (GEP-Q) and the entries in the reference database (GEP-DB) were based on pre-processed gene expression intensity values sub-setted to the 150 most up- and down-regulated genes from the vorinostat treatment of SKB cells. The drug vorinostat is a small molecule inhibitor of histone deacetylases (HDACs). Pharmacologically, it is used as antineoplastic agent and to treat cutaneous T-cell lymphomas (CTCL). It was chosen for this proof-of-concept test because several related HDAC inhibitor drugs with well annotated target annotations are represented in the LINCS database. Moreover, it has been used for similar reasons by other benchmark studies (1) to determine whether GESs of structurally and mechanistically related drugs are able to enrich each other at the top of GESS results.

**Figure 5.**
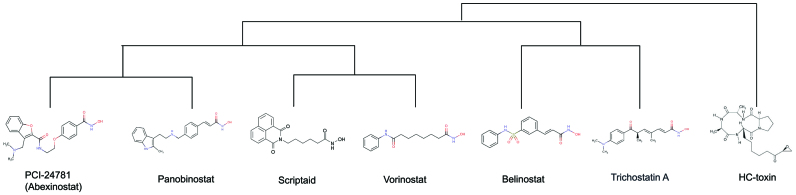
Structure-based hierarchical clustering dendrogram for drugs listed in Table 4. Experimental drugs lacking structure information are not included.

Table 4 shows the top ten drugs of the vorinostat GESS result identified by *SPsub* and ranked by absolute correlation coefficients. Impressively, nearly all of the top ranking drugs are annotated to target the same or similar HDACs as the vorinostat query. Most importantly, the remaining two drugs in the table, KM-00927 and PCI-24781 (Abexinostat), are not yet annotated as HDAC inhibitors in the corresponding drug-target databases. However, two recent studies have identified them as novel HDAC inhibitors (43,44). PCI-24781 is an experimental drug candidate for cancer treatment, that has been approved for Phase II clinical trials for the treatment of B-cell lymphoma. It has also been identified as a novel hydroxamic acid-based HDAC inhibitor (45). This result is an excellent example for demonstrating the power of the GESS technology in identifying targets for experimental drugs, as well as novel targets for drug repurposing approaches. Figure 5 compares the corresponding chemical structures of the compounds listed in Table 4. They are plotted in the order of a hierarchical clustering dendrogram generated with the structure-based clustering utilities of the affiliated *ChemmineR* package (27). While it is not expected that GESS-based rankings will perfectly agree with structure-based rankings, at least in this case the compound groupings of the two methods are in reasonable agreement, as several compounds in Table 4 are indeed structurally related, such as PCI-24781, panobinostat, scriptaid and vorinostat.

**Table 4.** Top ranking drugs of vorinostat query

Rank^a^	Drug name^b^	Cell type^c^	SCC^d^	Targets^e^
1	Vorinostat	SKB	1.00	HDAC1; HDAC10; HDAC11...
2	Trichostatin-a	SKB	0.99	HDAC1; HDAC10; HDAC2...
3	KM-00927	SKB	0.98	
4	Scriptaid	SKB	0.97	HDAC1; HDAC2; HDAC3...
5	HC-toxin	SKB	0.97	HDAC1
6	Belinostat	SKB	0.97	HDAC1; HDAC10; HDAC11...
7	Panobinostat	SKB	0.96	HDAC1; HDAC10; HDAC11...
8	PCI-24781	ASC	0.95	
9	HC-toxin	ASC	0.95	HDAC1
10	Vorinostat	ASC	0.94	HDAC1; HDAC10; HDAC11...

The GES of SKB cells treated with vorinostat was used as query to search the LINCS database with the *SPsub* method. The rows are sorted decreasingly by absolute Spearman Correlation Coefficients^d^. The other columns include ranks^a^, drug names^b^, cell types ^c^ and the gene symbols of the corresponding target sites^e^.

Next, the top 100 drugs of the vorinostat GESS result were functionally annotated with the FEA methods implemented in *signatureSearch*. Since the results of the different FEA methods were similar, the following considers only the results of the *dup_hyperG* method. Table 5 shows the five highest ranking GO terms of the Molecular Function (MF) and Biological Process (BP) ontology. The most highly enriched terms of the MF ontology are all related to histone deacetylase activity. This is expected since the target sites of the top ranking drugs are predominantly HDACs. The corresponding enrichment result for the BP ontology agrees well with the MF result since it is also dominated by histone deacetylation processes. Given vorinostat’s HDAC inhibitor activity, the obtained FEA results demonstrate the efficiency of *signatureSearch’s* FEA methods in identifying the correct pathways targeted by a query drug. Besides processes related to histone deacetylase activities, several biologically connected processes are enriched as well (Table 5), such as hair follicle placode formation. This is interesting because a recent study has shown that the suppression of epidermal HDAC activity leads to disrupted hair follicle regeneration and homeostasis (46). This finding demonstrates the utility of the GESS/FEA workflow in identifying alternative target pathways that may enable novel drug repurposing approaches for query drugs of interest in the future. To highlight the importance of the FEA step in the overall workflow, Supplementary Table S1 provides the enrichment results when using the genes of the initial GEP-Q instead of the downstream drug-target gene set from the GESS result for the same GO term enrichment analysis. When comparing the top ranking GO terms in both Table 5 and S1 then there are no top ranking GO terms shared among the results. This is not surprising since the GES-Q contains the genes exhibiting the most pronounced expression changes after treating SKB cells with vorinostat, while the genes used for the FEA analysis are the genes encoding the target proteins of the top ranking drugs in the initial GESS search result. Typically, there are no or only minor overlaps expected among the genes in these two sets (here 1.6% of GEP-Q). Most importantly, only the FEA approach identifies the correct target pathway for the vorinostat query, whereas the GO term enrichment analysis with the genes from the initial GES-Q contains terms that are fundamentally different and unrelated to the vorinostat target pathway. This comparison demonstrates the critical role of the FEA method for the overall analysis workflow in predicting target pathways in drug-based GESS results with *signatureSearch*.

**Table 5.** Top ranking MF and BP terms obtained with *dup_hyperG*

Ontology^a^	GO term^b^	*N* GO^c^	*N* test^d^	*N* match^e^	*P*-value^f^	*P*-adjust^g^
MF	HDAC activity (H3-K14) (GO:0031078)	11	323	97	0.00e+00	0.00e+00
MF	NAD-dependent HDAC activity (H3-K14, GO:0032041)	11	323	97	0.00e+00	0.00e+00
MF	NAD-dependent HDAC activity (GO:0017136)	16	323	98	0.00e+00	0.00e+00
MF	NAD-dependent PDAC activity (GO:0034979)	17	323	99	0.00e+00	0.00e+00
MF	HDAC activity (GO:0004407)	44	323	98	0.00e+00	0.00e+00
BP	Histone H3 deacetylation (GO:0070932)	21	323	98	0.00e+00	0.00e+00
BP	Histone H4 deacetylation (GO:0070933)	11	323	59	0.00e+00	0.00e+00
BP	Histone deacetylation (GO:0016575)	86	323	101	0.00e+00	0.00e+00
BP	Hair follicle placode formation (GO:0060789)	5	323	23	0.00e+00	0.00e+00
BP	Fungiform papilla morphogenesis (GO:0061197)	5	323	23	0.00e+00	0.00e+00

The columns contain: GO ontology^a^; GO term description/ID^b^; number of proteins in GO term^c^, test set^d^ and intersect^e^, raw *P*-value^f^, and adjusted *P*-value^g^ using the BH method for multiple testing correction. To save space, longer GO term descriptions have been shortened.

**Figure 6.**
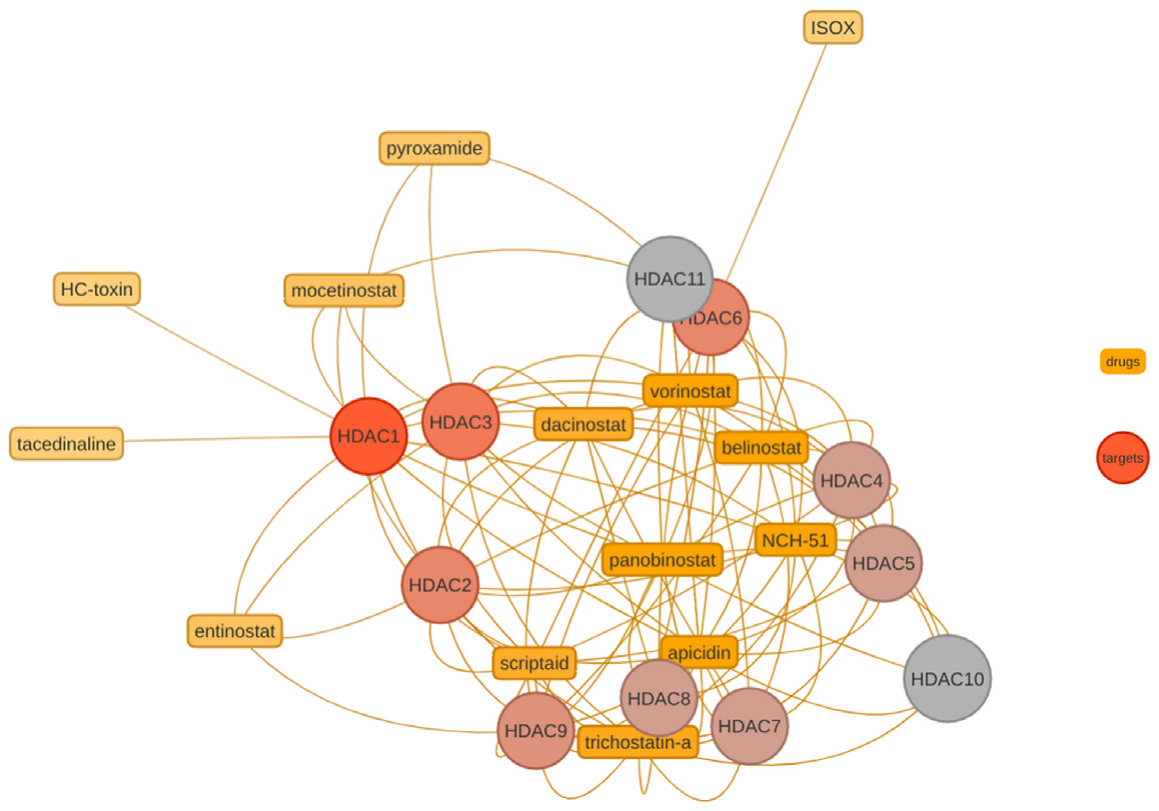
Drug-target network module of Histone Deacetylase Activity (H3-K14 specific; GO MF ID: GO:0031078). Drugs and targets are depicted as boxes and circles, respectively. The color of the circles indicates the number of connections.

Subsequently, drug-target networks (DTNs) were constructed to visually interpret the FEA results, and to prioritize interesting candidate drugs. A sample DTN is shown in Figure 6 where the term ’histone deacetylase activity’ (H3-K14 specific; GO:0031078) was chosen since it is one of the highest scoring GO MF terms in the result of the previous workflow step. The drugs and target proteins are depicted in Figure 6 as yellow boxes and circles, respectively, including vorinostat and its histone deacetylase targets. In the *signatureSearch* package these DTN graphs are fully interactive, where users can zoom into network modules, as well as select drugs and/or proteins in the drop-down menu located in the upper left corner of the plot.

## DISCUSSION

We have developed *signatureSearch* as an integrated and extendable environment for performing GESSs with a variety of algorithms combined with FEA and DTN visualization methods. The latter two are useful for guiding the downstream biological interpretation of GESS results. As outlined in the introduction and method sections, the software provides many useful and unique features, such as access to an end-to-end workflow toolkit covering most functionalities required for a wide range of GESS applications relevant to discovery-oriented research. It also provides access to an unmatched number of algorithms for both GESS and FEA routines, where we introduce several novel enrichment algorithms for interpreting GESS results. Importantly, the GESS methods in *signatureSearch* scale from single GES queries to large scale applications with thousands of GES queries using public or custom reference databases. This enables permutation tests with large numbers of randomized queries required to evaluate the robustness of GESS/FEA results. Typically, these types of large scale queries are not practical to support in other GESS tools that are predominantly based on web services.

This study is also unique by testing the performance of the GESS algorithms in recalling MOA and SSC categories with drug-induced query GESs. To the best of our knowledge, the performance of GESS methods has not been systematically compared as it has been done here. In these performance tests we find that the correlation-based methods, *SPall* and *SPsub*, outperform most set-based methods with respect to the chosen ROC performance criteria. Among the set-based methods LINCS performs the best, most likely because of the additional weighting information utilized by its algorithm.

Although correlation-based GESS methods show the best performance in our tests, the query types required for them are more complex than the simple gene identifier sets required for the set-based methods. Moreover, for compatibility reasons the quantitative queries of correlation methods should preferentially be derived from the same gene expression technology and organism used for generating the reference database. In this regard, the set-based methods are less restrictive and more versatile than correlation-based methods. Especially, for complex expression experiments, it is often easier to obtain a GES query composed of an identifier set of induced and repressed genes than the quantitative counterpart required for correlation-based approaches. Query gene sets from related species can also be used by translating them via ortholog mappings to the corresponding genes represented in the GES database. Moreover, set-based methods are more likely to exhibit reasonable performance in cross-omics queries, such as querying transcriptomic GES databases with up- and down-regulated gene sets from GWAS, proteomics or possibly even metabolomics studies. In summary, an advantage of set-based methods is that they are more technology agnostic but may not reach the recall performance of correlation-based methods.

Integrating important GESS and FEA methods into an R/Bioconductor package also offers several unique advantages not present in related software applications. Here, the *signatureSearch* packages simplifies the development of automated end-to-end workflows for conducting signature searches in many application areas. It consolidates an extendable number of GESS and FEA algorithms into a single environment that allows users to compare results among methods as well as define and incorporate custom methods. Moreover, the usage of generic data objects and classes improves maintainability and reproducibility of the provided functionalities, while the integration with the existing R/Bioconductor ecosystem maximizes their extensibility and reusability for other data analysis applications. Finally, *signatureSearch* provides access to several community perturbation reference databases along with options to build custom databases with support for most common mRNA expression profiling technologies. This design will also support expression profiling databases from other omics domains such as proteomics.

## CONCLUSION

*signatureSearch* provides a general purpose environment for identifying similar GESs in reference databases, while also guiding the downstream functional interpretation of the discovered connections. The functionalities of the package pave the way for discovering biologically relevant connections in gene networks. Those are useful to gain insights into stress-response pathways, to improve treatments for diseases, or to identify novel target site candidates for experimental drug-like small molecules or alternative targets of approved drugs for drug-repurposing approaches. In the future we will continue to enhance the package by adding several new features. First, we will include additional GESS/FEA methods optimized and tested for sparse GES data, such as single cell experiments. Second, support will be added for managing large numbers of heterogeneous query GESs in a single container that can be populated from flat files or a custom query database. Third, a batch run function will be added to execute the GESS/FEA workflow on any number of these heterogeneous queries automatically. Fourth, support for community workflow environments, such as CWL and *systemPipeR* (47), will be added to operate *signatureSearch* from start to finish from R or other popular programming languages such as Python or Bash.

## AVAILABILITY OF SOFTWARE AND DATA

*signatureSearch* and *signatureSearchData* are open source packages that have been reviewed, tested and accepted by the Bioconductor project. Both are freely available for all common operating systems from Bioconductor and GitHub here: https://bioconductor.org/packages/signatureSearch and https://bioconductor.org/packages/signatureSearchData.

## Supplementary Material

gkaa878_Supplemental_File
