# Supplementary material for: signatureSearch: environment for gene expression signature searching and functional interpretation
Source: Nucleic Acids Res. 2020 Oct 17;48(21):e124. doi: 10.1093/nar/gkaa878 (PMC7708038; doi:10.1093/nar/gkaa878)
Supplement: gkaa878_Supplemental_File [file gkaa878_supplemental_file.pdf]

SUPPLEMENTARY MATERIAL

SUPPLEMENTARY SECTIONS

S1

Pre-processing and cutoffs for queries and databases

The quantitative gene expression profiling (GEP) data used by this study were downloaded from Bioconductor's ExperimentHub with utilities provided by the *signatureSearchData* package. The latter provides pre-configured data sets for this project. At the time of writing, the GEP databases (GEP-DBs) included in *signatureSearchData* are LINCS and CMAP2 (1, 2). Since the experiment section of this article uses LINCS data, the following focuses on the pre-processing and filtering routines of this dataset. The non-quantitative gene sets (GSs) used as GS queries (GS-Qs) and GS databases (GS-DBs) in the article were also extracted from LINCS. The corresponding filtering parameters for obtaining these GSs are given in the next paragraph. Similar information, with additional details for both LINCS and CMAP2, is available in the vignette of the *signatureSearchData* package. Although CMAP2 was not used in the experiment section of this article, the following does include an overview of the corresponding pre-processing routines of this data set mainly to illustrate how to use CMAP2 instead of LINCS for similar analyses.

LINCS GEP data. The Broad Institute has generated the LINCS GEP data with the bead-based L1000 assay for gene expression profiling. Since this technology is not widely used yet and pre-processing methodologies for its data are limited in the public domain, we have chosen to use the pre-generated data instances from the LINCS project directly rather than attempting to regenerate them from raw data. The GEP data from LINCS data can be downloaded from GEO in 5 different pre-processing levels (2). Level 1 data are the raw mean fluorescent intensity values that come directly from the Luminex scanner. Level 2 data are the expression intensities of the 978 landmark genes. They have been normalized and used to impute the expression of an additional set of 11,350 genes, forming Level 3 data. A robust Z-scoring procedure was used to generate differential expression values from the normalized profiles (Level 4). Finally, a moderated Z-scoring procedure was applied to the replicated samples of each experiment (mostly 3 replicates) to compute a weighted average signature (Level 5). For a more detailed description of LINCS' pre-processing methods, readers want to refer to the methods section in the corresponding publication by Subramanian *et al.*, 2017 (2).

The differential expression data from LINCS used in this article are level 5 Z-scores. Since some GESS methods such as *gCMAP* and *Fisher* require gene sets in the reference database, Z-score cutoffs can be used to filter for sets of up- and down-regulated differentially expressed genes (DEGs). In this article, the corresponding up or down DEG sets were obtained with Z-score cutoffs of ≥ 1 or ≤ -1, respectively. In *signatureSearch*, these Z-score cutoffs can be assigned to filtering arguments to generate either query or database instances meeting the corresponding Z-score constraints. Examples of GS-DBs where this is relevant are those used by the *gCMAP* and *Fisher* GESS methods. In addition to using Z-score cutoffs, GS-Qs can also be extracted by specifying a fixed number of the most extremely up- and down-regulated genes, such as the top 150 up- and down-regulated DEGs, respectively. Whether GS-Qs or GS-DBs instances were obtained by Z-score or number cutoffs is specified in the corresponding sections of the article. If the cutoff parameters deviate from the above default values then they are given as well. Examples of GESS function calls related to these routines are provided in the vignettes of the software and data packages of the *signatureSearch* environment. For instance, the subsection '*DEG and Cutoff Definitions*' in the *signatureSearchData* vignette provides details on this topic.

CMAP2 GEP data This section provides a short overview of the CMAP2 data pre-processing steps to illustrate how this drug-perturbation GEP-DB could be used instead of LINCS for the performance test and proof-of-concept experiments included in this article. Both databases are supported by *signatureSearchData*, but for consistency we only used the LINCS database in the experimental sections. Since the Affymetrix GeneChip® technology used by CMAP2 is supported by a rich ecosystem of widely used analysis software, we generated the pre-processed and final data tables for this data set from the corresponding raw files (here CEL files), and deposited the results on Bioconductor's ExperimentHub for easy access with *signatureSearchData*. To compare the search results generated with the CMAP2 online service and the GESS methods from *signatureSearch*, we also included the CMAP2 rank matrix that is based on rank transformed differential expression values for all assayed genes. The latter can be downloaded from the CMAP2 project site. For the raw data processing from CEL files, normalized gene expression data were generated with the MAS5 algorithm (3). Next, the DEG analysis was performed with the *limma* package (4) using the experimental design table included in the CMAP2 data set to define replicates, as well as control and treatment samples. The statistical result tables generated by *limma*, including LFC values, p-values and false discovery rates (FDR), were saved to the HDF5 files we are hosting on Bioconductor's ExperimentHub. These statistical values can be used by the query retrieval and GESS methods in *signatureSearch* to define DEGs with single or combinatorial cutoff parameters, such as DEGs that have an LFC value of ≥ 1 or ≤ -1, and an FDR of ≤ 0.01. Although the LINCS and CMAP2 result tables had to be generated with different statistical methods, one can filter in both cases for DEGs with cutoffs that can be applied to statistical values with comparable meaning (*e.g.* LFCs can be used instead of Z-scores). Detailed instructions along with the corresponding R code for creating the corresponding gene expression and statistical result tables are provided in the CMAP2 pre-processing sections of the *signatureSearchData* vignette. For instance, instructions for defining DEG sets with combinatorial filters of statistical parameters are given in the Supplement section of the vignette under '*DEG and Cutoff Definitions*'.

S2

Additional details about FEA algorithms

Duplication Adjusted Hypergeometric Test (dup_hyperG). The classical hypergeometric test assumes uniqueness in its gene/protein test sets. Its p-value is calculated according to

$$p=\sum_{k=x}^{n}\frac{\binom{D}{k}\binom{N-D}{n-k}}{\binom{N}{n}}. \quad (\mathbf{1})$$

In case of GO term enrichment analysis the individual variables in equation (**1**) are assigned the following values. N is the total number of genes/proteins contained in the entire annotation universe, D is the number of genes annotated at a specific GO node, n is the total number of genes in the test set, and x is the number of genes in the test set annotated at a specific GO node. To maintain the duplication information in the test set used for TSEA, the values of n and x in the above equation are the corresponding gene counts including duplications.

Modified Gene Set Enrichment Analysis (mGSEA). The original GSEA method (5) uses predefined gene sets Ss defined by a chosen functional annotation system, such as GO or KEGG categories. The goal is to determine whether the genes in S are randomly distributed throughout a ranked test gene list L (*e.g.* all genes ranked by LFC), or enriched at the top or bottom of L. This is expressed by an Enrichment Score (ES) reflecting the degree to which a set S is overrepresented at the extremes of L. For TSEA, the test set L is a target set T associated with the top ranking drugs in a GESS result obtained from a drug-based GES database. Frequently, the corresponding gene identifiers in T are not unique, because several drugs in a GESS result may share the same targets. To account for the characteristic nature of GESS results, it is of utmost importance to maintain this duplication information as much as possible. To perform GSEA with duplication support, here referred to as *mGSEA*, the target set T is transformed to a score ranked target list L_{tar} of all targets included in the corresponding annotation system. For each target in T, its frequency is divided by the number of all targets in T (including duplications), which is the weight of that target. For targets present in the annotation system but absent in the target set T, their scores are set to 0. Thus, every target in the annotation system will be assigned a score. Subsequently, the target list will be sorted decreasingly to obtain L_{tar}. Importantly, the original GSEA method cannot be used for TSEA directly since zeros are very frequent in L_{tar}. As a result, the sum N_R can become zero too which cannot be used as the denominator in equation (**2**) from Subramanian et al. (2005). To avoid this problem, the affected ES values are ignored by assigning -1 as a tag.

$$P_{\text{hit}}(S,i)=\sum_{\substack{g_j\in S\\ j\le i}}\frac{|r_j|^p}{N_R}, \quad \text{where } N_R=\sum_{g_j\in S}|r_j|^p \quad (\mathbf{2})$$

If only some genes in set S have scores of zeros then the value of N_R is increased according to equation (**3**). The latter adds to N_R the minimum value of the non-zero gene scores in S multiplied by the number of genes in S that have scores of zero. Increasing N_R can in return decrease the weight of the genes in S that have non-zero scores. To compensate for this, the *mGSEA* algorithm computes N_R according to equation (**3**) instead of equation (**2**). $P_{\text{hit}}(S,i)$ in equation (**2**) evaluates the fraction of genes in S ("hits") weighted by their scores present up to a given position i in L_{tar}, where r_j is the score of gene j in L_{tar}. Typically, the exponent p is set to 1 in order to weight the genes in S by their scores in L_{tar}.

$$N_R=\sum_{g_j\in S}|r_j|^p+min(r_j|r_j>0)*\sum_{g_j\in S}I_{r_j=0} \quad (\mathbf{3})$$

The motivation for the above modifications is that if only a small number of genes in set S has non-zero scores and these genes rank high in L_{tar}, the weight of these genes will be close to 1 resulting in an $ES(S)$ of close to 1. Thus, the original GSEA method would score the gene set S of a functional category as significantly enriched. However, this is undesirable because in this example only a small number of genes is shared among the test target set T and the gene set S of a functional category. To avoid this, small weights are assigned to genes in S that have scores of zero. The latter decreases the weight of the genes in S that have scores other than zero, thereby decreasing the false positive rate. Finally, the functional categories (gene sets Ss) are ranked by ES from highest to lowest, where the top ranking ones are favored as enriched GO terms and KEGG pathways.

MeanAbs (mabs). The input for the *MeanAbs* method is L_{tar}, the same as for *mGSEA*. In this enrichment statistic, $mabs(S)$, of a gene set S is calculated as mean absolute scores of the genes in S (6). In order to adjust for size variations in gene set S, random permutations (*e.g.* $\pi=1000$) of L_{tar} are performed to determine $mabs(S,\pi)$. Next, $mabs(S)$ is normalized by subtracting the median of the $mabs(S,\pi)$ and then dividing by the standard deviation of $mabs(S,\pi)$ yielding the normalized scores $Nmabs(S)$. Subsequently, the portion of $mabs(S,\pi)$ that is greater than $mabs(S)$ is used as nominal p-value. Finally, the resulting nominal p-values are adjusted for multiple hypothesis testing using the Benjamini-Hochberg method (7).

S3

Filtering of MOA and SSC Categories

The 276 MOA categories were downloaded from the Touchstone database. They were associated with at total of 1,555 compounds. Since not all MOA categories are expected to contain drugs that induce similar gene expression changes, MOA categories predominantly associated with dissimilar GESs were eliminated by a filtering process based on recall rates that were averaged across all six GESS methods. For this, the GESs associated with drugs belonging to a MOA category were searched iteratively against the LINCS database. For each query result, the rankings of the GESs belonging to

the same MOA category as the query were recorded. The joined ranking results for all queries of a MOA were then summarized using the mean of the ranks, and the mean rank percentile was set as the recall rate of a MOA for the corresponding GESS method. To make sure none of the six GESS methods had been given an unfair advantage in this selection process, the MOA level recall rates were combined by calculating the mean of the recall rates across all six GESS methods. The latter was used for the final ranking of the MOA categories. Subsequently, the top 25% ranking MOA categories were used for the GESS performance tests described in the main text of this article. The final set included a total of 69 MOA categories associated with 309 compounds. The filtering of the SSC categories was performed the same way as the filtering of the MOA categories.

SUPPLEMENTARY TABLES

Table S1: Top GO Terms with GES Query

Table S1. Top ranking GO MF and BP terms obtained from direct enrichment of the vorinostat GS-Q with hypergeometric test. The columns contain: GO Ontology[a]; GO Term description/ID[b]; number of genes in GO term[c], test set[d] and intersect[e], respectively; as well as enrichment p-value[f] and adjusted p-value[g] using the Benjamini-Hochberg (BH) method. To save space, longer GO term descriptions have been shortened.

Ontology[a]	GO Term[b]	N GO[c]	N Test[d]	N Match[e]	P-Value[f]	P-Adjust[g]
MF	phospholipase activator activity (GO:0016004)	12	295	4	3.4e-05	0.013
MF	kinase regulator activity (GO:0019207)	207	295	13	4.6e-05	0.013
MF	lipase activator activity (GO:0060229)	14	295	4	6.6e-05	0.013
MF	transcription coactivator activity (GO:0003713)	319	295	16	9.6e-05	0.014
MF	RNA polymerase II TF binding (GO:0001085)	155	295	10	2.8e-04	0.024
BP	cellular response to peptide (GO:1901653)	385	293	21	8.3e-07	0.003
BP	regulation of apoptotic signaling pathway (GO:2001233)	406	293	20	7.1e-06	0.010
BP	histone modification (GO:0016570)	454	293	21	1.1e-05	0.010
BP	response to metal ion (GO:0010038)	364	293	18	1.9e-05	0.010
BP	covalent chromatin modification (GO:0016569)	474	293	21	2.1e-05	0.010

Table S2: Statistical Tests for Performance Differences Among GESS Methods Applied to MOA Categories

Table S2. GESS methods applied to MOA categories. To assess whether the observed performance differences are statistically significant for all pair-wise comparisons, the bootstrap method was used for both the global AUC and pAUC metrics. The BH method was used for multiple testing correction (7). The columns contain: GESS method 1[a]; GESS method 2[b]; P-value[c] and adjusted P-value[d].

		AUC		pAUC (FPR 0.01)		pAUC (FPR 0.05)		pAUC (FPR 0.10)	
GESS1[a]	GESS2[b]	P-Value[c]	P-Adjust[d]	P-Value	P-Adjust	P-Value	P-Adjust	P-Value	P-Adjust
gCMAP	CMAP	6.2e-70	1.3e-69	2.7e-11	2.9e-11	4.7e-29	5.4e-29	1.3e-39	1.5e-39
gCMAP	Fisher	1.4e-104	3.5e-104	1.3e-56	2.7e-56	1.6e-96	4.0e-96	1.1e-124	3.2e-124
gCMAP	SPall	8.1e-217	6.1e-216	9.8e-44	1.6e-43	3.7e-72	6.2e-72	2.9e-89	5.4e-89
gCMAP	LINCS	3.0e-182	1.5e-181	1.6e-97	4.9e-97	1.6e-193	2.4e-192	2.0e-211	1.5e-210
gCMAP	SPsub	7.0e-236	1.0e-234	6.4e-145	9.6e-144	3.3e-181	2.5e-180	8.9e-218	1.3e-216
CMAP	Fisher	1.7e-27	2.1e-27	1.1e-48	2.1e-48	1.0e-60	1.5e-60	3.2e-69	4.7e-69
CMAP	SPall	5.3e-65	9.9e-65	5.4e-38	8.1e-38	2.0e-37	2.5e-37	7.3e-42	9.1e-42
CMAP	LINCS	1.5e-132	5.8e-132	4.0e-86	1.0e-85	1.1e-151	5.3e-151	2.7e-159	1.3e-158
CMAP	SPsub	2.0e-128	5.9e-128	2.2e-125	1.6e-124	1.7e-144	6.3e-144	2.4e-141	8.8e-141
Fisher	SPall	1.2e-04	1.3e-04	1.5e-07	1.5e-07	9.1e-19	9.8e-19	1.4e-15	1.5e-15
Fisher	LINCS	2.8e-28	3.8e-28	3.9e-13	4.5e-13	7.8e-60	1.1e-59	4.4e-62	6.1e-62
Fisher	SPsub	2.3e-62	3.9e-62	2.3e-99	1.1e-98	1.2e-84	2.2e-84	7.6e-75	1.3e-74
SPall	LINCS	1.4e-16	1.6e-16	2.8e-22	3.4e-22	1.1e-85	2.4e-85	1.1e-90	2.3e-90
SPall	SPsub	1.1e-49	1.6e-49	9.1e-99	3.4e-98	1.1e-105	3.2e-105	1.3e-116	3.3e-116
LINCS	SPsub	1.0e-01	1.0e-01	5.2e-33	7.1e-33	5.7e-02	5.7e-02	1.8e-01	1.8e-01

Table S3: Statistical Tests for Performance Differences Among GESS Methods Applied to SSC Categories

Table S3. GESS methods applied to SSC categories. The column titles and content of this table are organized the same way as in Table S2.

		AUC		pAUC (FPR 0.01)		pAUC (FPR 0.05)		pAUC (FPR 0.10)	
GESS1	GESS2	P-Value	P-Adjust	P-Value	P-Adjust	P-Value	P-Adjust	P-Value	P-Adjust
gCMAP	CMAP	7.7e-183	1.9e-182	1.3e-10	1.4e-10	8.7e-28	1.0e-27	5.6e-34	6.0e-34
gCMAP	Fisher	2.0e-155	3.8e-155	8.1e-64	1.3e-63	9.6e-147	1.6e-146	1.6e-197	3.0e-197
gCMAP	SPall	0.0e+00	0.0e+00	1.6e-59	2.4e-59	3.0e-96	4.0e-96	2.2e-130	3.0e-130
gCMAP	LINCS	0.0e+00	0.0e+00	2.7e-173	8.0e-173	0.0e+00	0.0e+00	0.0e+00	0.0e+00
gCMAP	SPsub	0.0e+00	0.0e+00	4.5e-236	3.4e-235	0.0e+00	0.0e+00	0.0e+00	0.0e+00
CMAP	Fisher	3.7e-28	4.3e-28	3.2e-54	4.4e-54	1.1e-114	1.6e-114	2.2e-135	3.2e-135
. CMAP	SPall	2.3e-84	3.2e-84	3.0e-52	3.8e-52	5.3e-64	6.7e-64	5.3e-78	6.6e-78
CMAP	LINCS	5.9e-190	1.8e-189	7.2e-166	1.8e-165	0.0e+00	0.0e+00	0.0e+00	0.0e+00
CMAP	SPsub	1.1e-275	4.0e-275	2.7e-251	4.1e-250	0.0e+00	0.0e+00	0.0e+00	0.0e+00
Fisher	SPall	1.3e-03	1.3e-03	8.2e-02	8.2e-02	6.9e-26	7.4e-26	3.8e-36	4.3e-36
Fisher	LINCS	4.6e-86	7.0e-86	8.0e-71	1.5e-70	7.2e-157	1.4e-156	1.0e-162	1.7e-162
Fisher	SPsub	7.7e-158	1.6e-157	7.3e-209	3.6e-208	1.1e-231	2.8e-231	1.1e-235	2.6e-235
SPall	LINCS	2.8e-53	3.5e-53	4.3e-72	9.3e-72	1.7e-185	3.7e-185	1.8e-200	3.9e-200
SPall	SPsub	2.5e-153	4.2e-153	5.1e-189	1.9e-188	8.2e-253	2.5e-252	1.4e-300	4.3e-300
LINCS	SPsub	3.8e-15	4.1e-15	3.1e-32	3.6e-32	7.9e-11	7.9e-11	1.0e-04	1.0e-04

REFERENCES

1. Lamb, J., Crawford, E. D., Peck, D., Modell, J. W., Blat, I. C., Wrobel, M. J., Lerner, J., Brunet, J. P., Subramanian, A., Ross, K. N., Reich, M., Hieronymus, H., Wei, G., Armstrong, S. A., Haggarty, S. J., Clemons, P. A., Wei, R., Carr, S. A., Lander, E. S., and Golub, T. R. (2006) The Connectivity Map: using gene-expression signatures to connect small molecules, genes, and disease. *Science,* **313**, 1929–1935.
2. Subramanian, A., Narayan, R., Corsello, S. M., Peck, D. D., Natoli, T. E., Lu, X., Gould, J., Davis, J. F., Tubelli, A. A., Asiedu, J. K., Lahr, D. L., Hirschman, J. E., Liu, Z., Donahue, M., Julian, B., Khan, M., Wadden, D., Smith, I. C., Lam, D., Liberzon, A., Toder, C., Bagul, M., Orzechowski, M., Enache, O. M., Piccioni, F., Johnson, S. A., Lyons, N. J., Berger, A. H., Shamji, A. F., Brooks, A. N., Vrcic, A., Flynn, C., Rosains, J., Takeda, D. Y., Hu, R., Davison, D., Lamb, J., Ardlie, K., Hogstrom, L., Greenside, P., Gray, N. S., Clemons, P. A., Silver, S., Wu, X., Zhao, W.-N., Read-Button, W., Wu, X., Haggarty, S. J., Ronco, L. V., Boehm, J. S., Schreiber, S. L., Doench, J. G., Bittker, J. A., Root, D. E., Wong, B., and Golub, T. R. (2017) A Next Generation Connectivity Map: L1000 Platform and the First 1,000,000 Profiles. *Cell,* **171**, 1437–1452.e17.
3. Pepper, S. D., Saunders, E. K., Edwards, L. E., Wilson, C. L., and Miller, C. J. (2007) The utility of MAS5 expression summary and detection call algorithms. *BMC Bioinformatics,* **8**, 273.
4. Ritchie, M. E., Phipson, B., Wu, D., Hu, Y., Law, C. W., Shi, W., and Smyth, G. K. (2015) limma powers differential expression analyses for RNA-sequencing and microarray studies. *Nucleic Acids Res.,* **43**, e47.
5. Subramanian, A., Tamayo, P., Mootha, V. K., Mukherjee, S., Ebert, B. L., Gillette, M. A., Paulovich, A., Pomeroy, S. L., Golub, T. R., Lander, E. S., and Mesirov, J. P. (2005) Gene set enrichment analysis: a knowledge-based approach for interpreting genome-wide expression profiles. *Proc. Natl. Acad. Sci. U. S. A.,* **102**, 15545–15550.
6. Fang, Z., Tian, W., and Ji, H. (2012) A network-based gene-weighting approach for pathway analysis. *Cell Res.,* **22**, 565–580.
7. Benjamini, Y. and Hochberg, Y. (1995) Controlling the false discovery rate - a practical and powerful approach to multiple testing. *J. R. Stat. Soc. Series B Stat. Methodol.,* **57**, 289–300.
